# The First Experience of Catheter Based Pulmonary Embolectomy in Iran: A New Horizon in Therapy of Massive Pulmonary Emboli

**Published:** 2011-11-01

**Authors:** J Kojuri, P Dehghani, M A Ostovan, A R Abdi, M J Zibaeenejad

**Affiliations:** 1 Department of Cardiology, Shiraz Cardiovascular Research Center, Shiraz University of Medical Sciences, Shiraz, Iran

**Keywords:** Catheter, Pulmonary embolectomy, Therapy, Iran

Dear Editor,

Pulmonary thromboembolism (PTE) is a relatively common and hazardous emergency that may lead to acute life-threatening but potentially reversible right ventricular failure. Three months mortality rate in patients with massive pulmonary embolism and a systolic blood pressure of <90 mmHg is approximately 50%, with most deaths occurring within the first few days after diagnosis.[1][2]

For practical purposes and risk markers useful for risk stratification in PTE can be classified into three groups including (i) Clinical markers such as hypotension and shock, defined as systolic blood pressure <90 mmHg or a drop of pressure ≥ 40 mmHg for >15 minutes if not caused by new onset arrhythmia, hypovolemia and sepsis, (ii) Markers of right ventricular (RV) dysfunction that is shown by RV dilatation, hypokinesia or pressure overload on echocardiography, RV dilatation on spiral chest computed tomography, elevated right heart pressure at right heart catheterization and brain natriuretic peptide (BNP) or N terminal Pro- BNP (NT-proBNP) elevation and (iii) Markers of myocardial injury like positive cardiac troponin T or I.[2][3]

In patients with massive PTE, systemic thrombolysis or surgical embolectomy in addition to anticoagulation can improve mortality with reversing the cardiogenic shock and right ventricular failure.[2][3][4][5] However, there are many patients that are not eligible for thrombolysis because of contraindications and also few tertiary care centers are providing emergency pulmonary thromboembolectomy for patients with massive PTE. The only alternative to thrombolysis or surgical embolectomy is percutaneous catheter thrombectomy.[6][7]

All patients with PTE were registered in Shiraz, CT scan and Doppler sonography of both extremities, and echocardiography were performed for all. Those with persistent BP less than 90 mmhg in spite of ionotrope use or drop of systolic pressure more than 40 mmhg were considered as the cases of hemodynamically significant PTE. Thrombolysis was started for those who were eligible to thrombolysis after consultant visit. Those with contraindication for thrombolysis were consulted for surgical thrombectomy and if they were rejected by surgeons were considered as eligible cases for catheter based embolectomy.

Complete pressure and saturation study and selective left and right pulmonary angiography before and after procedure were done in all patients. Through the 8 French sheath over 0.018 guide wire Aspirex (Straus) embolectomy catheter was used and procedure was continued till hemodynamic improvement was seen. Catheter embolectomy was performed for 7 patients with mean age of 61 years that 57% were female from February 2008 till August 2010. Two of patients had remaining clot in pulmonary while the procedure was stopped due to hemodynamic improvement. Inferior Vena Cava (IVC) filter Optease (Cordis) was inserted for all patients. In our registry, all patients developed hemodynamic improvement post procedure without any complications and 6 of them (88%) were discharged home (Table 1). The first case was expired due to deep coma post cerebrovascular accident.

**Table 1:** Data of 7 patients with massive PTE, undergone catheter embolectomy in Shiraz, Iran

**Patient**	**1**	**2**	**3**	**4**	**5**	**6**	**7**
Age (Year)	88	54	58	65	45	72	45
Site of clot in CT Scan	Saddle	Left	Left and right	Main	Diffuse	Main	Left and right
Blood Pressure Pre procedure (mmHg)	80	70	85	90	70	85	90
Inotrope use	+	+	+	+	+	+	+
Indication	Post CVA	Post Surgery	Trauma	Post Surgery	Cancer	Post Surgery	Post Surgery
Blood pressure Post procedure (mmHg)	110	98	140	160	95	135	110
PA Pressure Pre procedure (mmHg)	45	54	65	48	58	64	45
PA Pressure Post procedure (mmHg)	38	34	35	34	28	43	25
Angiographic evidence of complete clot removal	+	+	+	+	-	+	-
IVC filter use	+	+	+	+	+	+	+
Outcome	Expired	Discharged	Discharged	Discharged	Discharged	Discharged	Discharged

Thrombolysis was contraindicated in 30-50% of cases post-massive PTE.[8] One of the most precipitating factors for PTE is prior surgery which is one of the contraindications for thrombolysis, and as it seen in Table 1, it is the most common cause of PTE in our patients. Surgical embolectomy can be done only in less than 1% of patients with massive PTE.[9][10]

In a review of the available literature, the overall clinical success rate, defined as immediate hemodynamic improvement, was > 80% for catheter embolectomy, and mortality rate ranged from 0 to 25% for the various techniques.[7]

Catheter intervention is currently being performed in patients with acute PTE and in shock state that an increased bleeding risk precludes the administration of thrombolysis, and surgical thrombectomy is not rapidly available or feasible. More researches are needed to investigate about other indications of catheter thrombectomy.
